# Effectiveness of, access to and need for electroconvulsive therapy in forensic psychiatric hospitals: a survey in Germany and Switzerland

**DOI:** 10.1007/s00406-025-02044-6

**Published:** 2025-06-23

**Authors:** Matthias Besse, Michael Belz, Henning Hachtel, Alfred Simon, Dirk Hesse, Jürgen Müller, Aniela Friese, David Zilles-Wegner

**Affiliations:** 1 Department of Psychiatry and Psychotherapy, University Medical Center Göttingen, von-Siebold-Strasse 5, D-37075 Göttingen, Germany; 2 Department of Forensic Psychiatry, University Medical Center Basel, Wilhelm Klein-Strasse 27, Basel, CH-4002 Switzerland; 3 Academy for Ethics in Medicine Göttingen, Humboldtallee 36, D- 37073 Göttingen, Germany; 4 Hospital of Forensic Psychiatry Moringen, Mannenstrasse 29, D- 37186 Moringen, Germany; 5 Asklepios Forensic Psychiatric Hospital Göttingen, Rosdorfer Weg 70, D-37081 Göttingen, Germany; 6 Asklepios Forensic Psychiatric Hospital Ochsenzoll, Langenhorner Chaussee 560, 22419 Hamburg, Germany

**Keywords:** Electroconvulsive therapy (ECT), Forensic psychiatry, Questionnaire, Treatment-resistance, Capacity to consent

## Abstract

**Objective:**

Patients with treatment resistant schizophrenia (TRS) may benefit from electroconvulsive therapy (ECT). Although TRS is a frequent problem in forensic hospitals, ECT is rarely used in this setting. This study investigates the availability and implementation of ECT in forensic hospitals in Germany and Switzerland. Moreover, we collected anonymized clinical data of patients treated with ECT.

**Methods:**

A digital survey was sent to all forensic psychiatric hospitals in Germany and Switzerland. The questionnaire comprised general information (hospital structure, use of ECT) and an optional second part for patients treated with ECT during the last 12 months.

**Results:**

41 German and 4 Suisse hospitals responded, of which the majority stated to have the possibility to offer ECT. The estimated percentage of patients with ECT indication was 7.35% (360 patients) and 7.5% (13 patients), respectively. However, only 38 patients were actually treated with ECT over a period of 12 months. Slightly over 50% of the patients were responders according to the Clinical Global Impression Improvement Scale.

**Conclusion:**

Our study presents the largest population of patients with TRS treated with ECT in forensic hospitals to date. Compared to 2018, there was a marked increase in the proportion of patients for whom ECT was considered indicated. Patients treated with ECT experienced a reduction in both symptom severity and the need for restraints. The response rate aligns with matching data from non-forensic populations. In view of these promising results, prospective controlled observational studies are needed to further strengthen the evidence regarding the effectiveness of ECT in forensic populations.

## Introduction

The current situation of forensic psychiatric hospitals in Germany (Ger) has been the subject of intense medical and political debate, resulting from various media reports [1–4] about overcrowded wards with worrying conditions and increasing aggression towards medical staff [5, 6]. Over the last twenty years, the number of patients detained in forensic psychiatric hospitals in Ger has more than doubled, reaching over 13.000 in 2021 [7].This increase lead to the amendment of Sect. 64 of the German Criminal Code in 2023, aiming to reduce the number of delinquents allocated to forensic addiction treatment facilities [6]. Still, there seems to be a growing proportion of detained patients suffering from schizophrenia in forensic psychiatry [8]. Being the most common diagnosis in forensic psychiatric hospitals, schizophrenia is known to be associated with an increased length of stay, especially in the case of treatment-resistance [9, 10].

Electroconvulsive therapy (ECT) is an established and highly effective treatment for psychotic disorders, even in clozapine-resistant schizophrenic patients [11, 12]. While over the last years the availability and number of ECT treatments have increased significantly in general psychiatric hospitals in German speaking countries [13, 14], scientific data about the use of ECT in the context of forensic psychiatry is still scarce. In a recently published systematic review we could merely identify five case reports, one case series and no randomized controlled studies, reporting a total number of 13 patients (10 suffering from schizophrenia), treated with ECT in forensic settings [15]. First systematic data about the current state and estimated need for ECT in forensic populations were collected by our research group in 2018 by sending a questionnaire to all forensic psychiatric hospitals in Ger. Results were ambiguous: While approximately two thirds of the responding hospitals stated a medium or even high need for ECT and a total number of patients with appropriate indication of 257, only 32 patients (= 12.5%) actually received ECT. Structural aspects were the most frequent reasons for not using ECT in the respective settings [16]. Shortly after our data collection, the German Association for Psychiatry, Psychotherapy and Psychosomatics (DGPPN) published an updated S3-guideline for schizophrenia. This version included a new recommendation for the use of ECT in patients with antipsychotic treatment-resistance [17]. Following this new recommendation, it is to be expected that the frequency of indications for ECT in patients with schizophrenia should have increased significantly since 2019, both in general psychiatry and forensic settings.

The present study aims to investigate the frequency of indications, number of applied ECT treatments and estimated need for ECT in forensic psychiatry for the year 2022/23 in comparison to our previous study from 2018. Besides these variables, anonymized demographic and clinical data of patients with ECT (age, diagnosis, indication, effectiveness, side-effects, treatment parameters) were systematically collected from forensic psychiatric hospitals in Ger and Switzerland (Sui).

## Methods

For the purpose of data collection, a two-part digital questionnaire in German language was designed by our research group, using LimeSurvey (LimeSurvey GmbH Hamburg). The first part contained the following eight questions, concerning a period of 12 month before the survey:

1. Number of detained patients in the respective hospital.
2. Estimated effectiveness of ECT in the treatment of depression on a 11-point numeric scale with two anchors (0 = not effective at all, 10 = highly effective).
3. Estimated effectiveness of ECT in the treatment of schizophrenia on a 11-point numeric scale with two anchors (0 = not effective at all, 10 = highly effective).
4. Estimated percentage of patients with indication for ECT treatment in the respective hospital.
5. Estimated need for ECT in forensic psychiatry on a 11-point numeric scale with two anchors (0 = no need, 10 = urgently needed).
6. Possibility to treat patients with ECT in the respective clinic and possible obstacles.
7. Willingness to treat incapable patients refusing ECT in principle.
8. Number of patients with ECT over the last 12 month.

If a responding hospital reported at least one treatment with ECT during the last 12 months, the second part of the questionnaire asked for demographic and clinical details for each case: age, sex, diagnosis, indication for ECT, consent, number of ECT treatments, electrode placement, and side-effects. The response outcome was measured by the Clinical Global Impression Scale (global improvement: CGI-I; [18]), side-effects.

All 78 forensic psychiatric hospitals in Ger and six in Sui were invited by e-mail at the beginning of 2023 to answer an online version of the questionnaire. Four weeks after the invitation, a first reminder was sent via e-mail. After another four weeks, all non-replying hospitals were re-contacted.

IBM SPSS Statistics 29 (IBM Corp. Armonk, NY) was used to analyze data. For descriptive representation of numeric variables, we created means (M) and standard deviations (SD) along with frequencies/percentages for categorical variables.

## Results

Out of 78 forensic psychiatric hospitals in Ger, *n* = 41 participated in the questionnaire study (response rate 52.6%). Out of these, 36 provided information on the number of detained patients, resulting in a total of 5054. Most common diagnoses were addictive disorders (46.8%) and schizophrenia (41.4%). Five hospitals exclusively reported patients with addictive disorders.

Out of 6 forensic psychiatric hospitals in Sui, *n* = 4 participated (response rate 66.7%), reporting a total of 175 detained patients. Schizophrenia was the most common diagnosis by far (83.6%).

### Experiences in the application of ECT

On the numeric scale from 0 to 10 (0 = not effective at all, 10 = highly effective), the estimated mean effectiveness of ECT for the treatment of depression was 8.00 in Ger (*n* = 39; SD = 1.56; Med = 8.00) and 8.50 in Sui (*n* = 4, SD = 0.58; Med = 8.50). For the treatment of schizophrenia, it was rated 6.05 in Ger (*n* = 39, SD = 1.89; Med = 6.00) and 5.00 in Sui (Sui; *n* = 3, SD = 2.65; Med = 5.00).

In Ger, the majority of respondents reported to have own experience in the application of ECT in patients with depression (*n* = 26 out of 40, 65.0%) and schizophrenia (*n* = 25 out of 40, 62.5%) within their hospital. In Sui, *n* = 1 out of 4 (25.0%) respondents stated existing ECT experience in the application of ECT in patients with depression, and *n* = 1 out of 4 (25.0%) in patients with schizophrenia.

### Indication and need for ECT

**Fig. 1:**
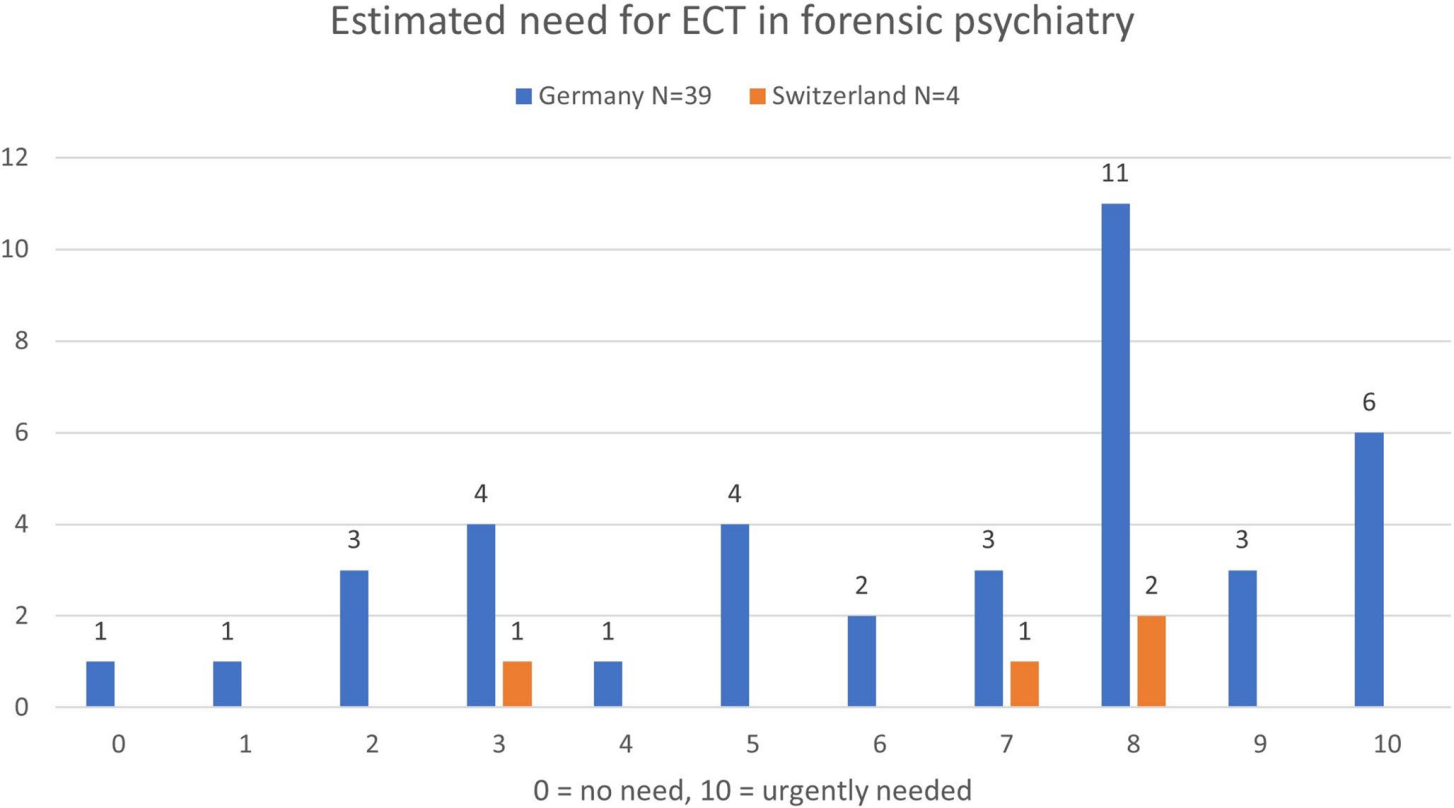
Estimated need for ECT in forensic psychiatry, rated by the participating forensic psychiatric hospitals from Germany and Switzerland

The respondents from Ger stated that M = 7.35% (*n* = 36, SD = 9.12, min = 0, max = 50; Med = 5.00) of the patients within their hospital had an indication for ECT. This resulted in a total number of 360 out of 4903 patients from the 36 hospitals that participated in this study. The general need for ECT in forensic psychiatry (see Fig. 1) was rated M = 6.44 (*n* = 39, SD = 2.88; Med = 8.00) on the numeric scale (0 = no need, 10 = urgently needed).

For the participating forensic hospitals from Sui, the respondents estimated that M = 7.50% (*n* = 4, SD = 5.00, min = 5, max = 15; Med = 5.00) of their patients had an indication for ECT, leading to a total number of 13 out of 175. The general need for ECT in forensic psychiatry (see Fig. 1) was rated 6.50 (*n* = 4; SD = 2.38, Med = 7.50).

### Possibility to perform ECT in the respective clinic

For *n* = 29 out of 41 forensic psychiatric hospitals in Ger (70.7%) and all *n* = 4 hospitals from Sui it was stated that they have the possibility to offer ECT. For the 12 clinics without ECT, the most common reason why it ECT could not be performed was described as lack of infrastructure (*n* = 9). Legal obstacles (*n* = 2) and ethical concerns (*n* = 1) were mentioned less frequently by the participants.

With regard to ECT in patients without capacity to consent, about half of the respondents from forensic psychiatric clinics with ECT stated that they would consider to treat patients against their will (Ger: *n* = 15, 51.7%; Sui: *n* = 2, 50.0%).

### ECT treatments over the last 12 months

For *n* = 13 forensic psychiatric hospitals in Ger (44.8% of the hospitals with possibility to perform ECT) participants reported at least one treatment with ECT over the last 12 months, with a total of 36 treated patients (min = 1, max = 8).

A total of 2 patients were treated in *n* = 1 forensic psychiatric hospital in Sui (25.0% of the hospitals with possibility to perform ECT).

The participating forensic psychiatric hospitals in Ger provided demographic and clinical data for *n* = 29 of the reported 36 patients, the Sui forensic psychiatric hospitals for both reported patients.

### Clinical data of patients with ECT

As described above, details were provided for *n* = 31 patients with ECT (Ger and Sui). All except one (ICD-10: F33.2) suffered from schizophrenia (ICD-10: F2x). The most common indication for ECT were treatment resistant psychotic symptoms (GER: *n* = 25 out of 29, 86.2%; Sui: *n* = 2 out of 2, 100%). Only one patient in Ger received ECT without consent, none in Sui.

**Fig. 2:**
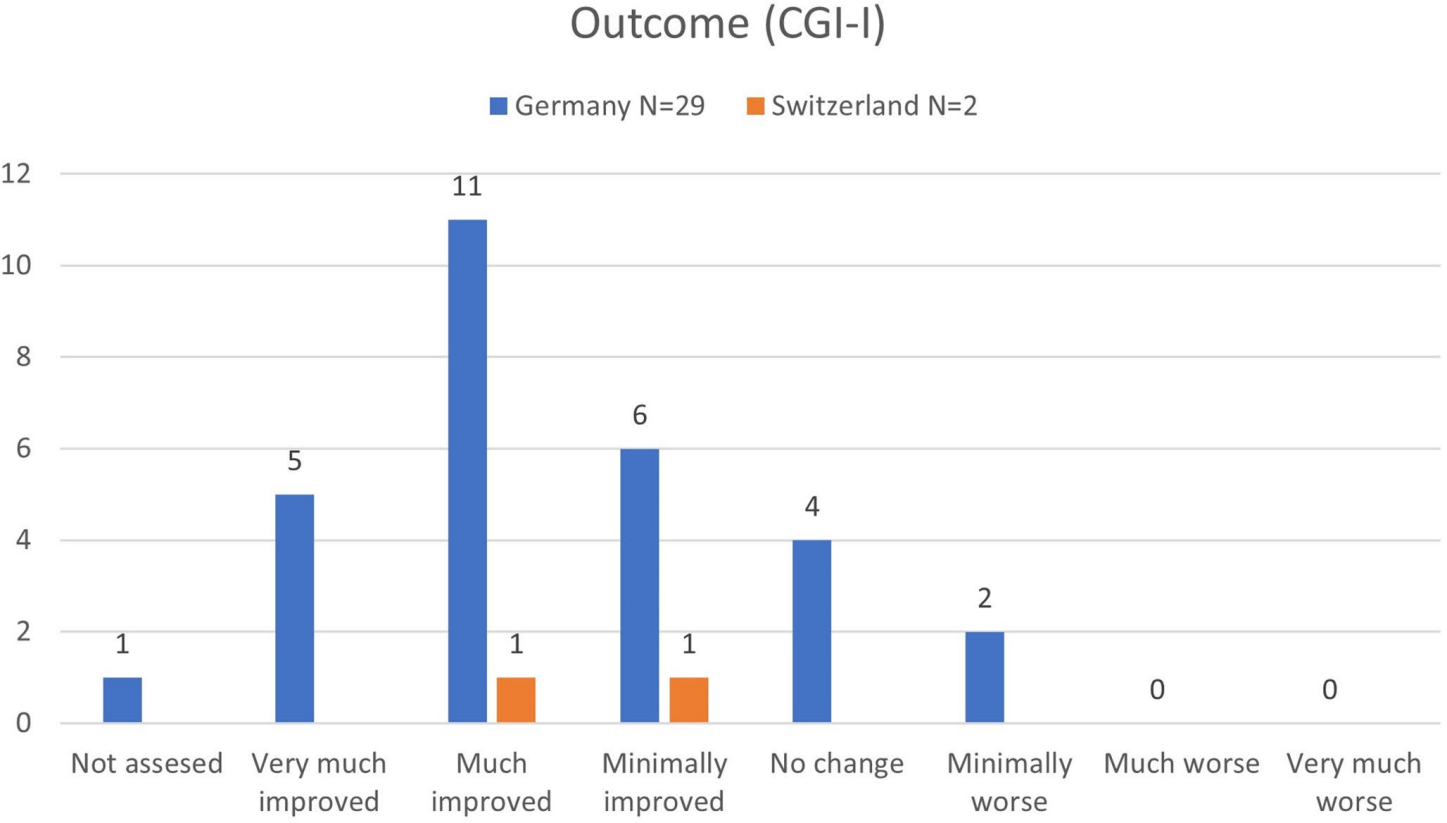
Reported outcome of the ECT-treated patients in German and Swiss forensic psychiatric hospitals, using the Clinical global impression scale– global improvement (CGI-I)

The majority of patients from Ger (*n* = 16 out of 29, 55.2%) and *n* = 1 out of 2 patients from Sui (50.0%) achieved a score of “1” or “2” on the CGI-I scale and thus could be identified as responders. In the subgroup of these *n* = 16 responders, restraints or detention could be reduced in 14 patients (87.5%) consequently. Furthermore, the majority of the patients with ECT in Ger (*n* = 16 out of 29, 55.2%) and both patients from Switzerland reported temporary side effects, specifically “cognitive decline” (Ger: *n* = 11 out of 29, 37.9%; Sui: *n* = 2 out of 2, 100%) and “post-ECT delirium” (Ger: *n* = 3 out of 29, 10.3%; Sui: no cases). For further details, please see Table 1; Fig. 2.

**Table 1:** Characteristics of ECT-treated patients

	Germany	Switzerland
Number of patients	29	2 (P1&P2)
Age (in years)	M = 40,43 (*N* = 28; SD = 10,95) min = 22, max = 66	P1 = 27, P2 = 43
Sex	Male = 28, Female = 1	P1 = Male, P2 = Female
Diagnosis (ICD-10)	F2x = 28, F33.3 = 1	F20.0 = 2
Indication for ECT	Treatment resistant psychotic symptoms = 25 Catatonia = 2 Depressive symptoms = 2	Treatment resistant psychotic symptoms = 2
Number of ECT treatments	M = 21,04, Med = 12 (*N* = 28; SD = 36,22) min = 2, max = 200	P1 = 19, P2 = 33
Electrode placement (multiple answers possible)	Unilateral = 23, LART = 19, bitemporal = 15, bifrontal = 1	bitemporal = 2
Consent	With consent = 24, without consent = 1 (*N* = 25)	With consent = 2
Side-effects	Side-effects occurred = 16 • Transient cognitive decline = 11 • Delirium = 3 • Other = 2	Side-effects occurred = 2 • Transient cognitive decline = 2
Outcome (CGI-I)	M = 2,45 (SD = 1,24) (see Fig. 2)	P1 = 3, P2 = 2 (see Fig. 2)
Response (CGI-I = 1 or 2)	Yes = 16; No = 12; Not assessed = 1	P1 = No; P2 = Yes
Outcome (relaxation of confinement)	Yes = 17, No = 6 (*N* = 23)	P1 = No, P2 = Yes

Demographic and clinical details for ECT-treated patients in Germany and Switzerland. M=mean value; Med=median; SD=standard deviation; min=minimum; max=maximum; LART=left anterior right temporal; N=number; P1=patient 1; P2=patient 2

## Discussion

This is the first study to systematically collect clinical data of patients treated with ECT in forensic psychiatric hospitals in Ger and Sui. Furthermore, we investigated the frequency of indications, number of applied ECT treatments and estimated need for ECT in forensic psychiatry in both countries. Although the scope and thus also the length of the questionnaire has increased significantly in comparison to our first study of 2018, we received answers from 41 (Ger) and 4 (Sui) forensic psychiatric hospitals, which corresponds to a reply rate of 52.6% (Ger) and 66.7% (Sui), respectively. These relatively high rates might reflect the importance of the topic ECT in the context of forensic psychiatry and the necessity for further scientific studies. Compared to our previous study of 2018 [16], the most important findings are (1) a doubled indication rate for ECT treatment, (2) a higher estimated need for ECT in forensic psychiatry, and (3) response rates comparable to those in general psychiatry (> 50.0%). The overall number of ECT-treatments can still be considered low.

The estimated quantity of patients with an indication for ECT-treatment has more than doubled compared to our last study [16] (Ger: 2018 = 3.4%; 2024 = 7.35%). It is ten times (Ger) or 6.5 times (Sui) higher than the number of actually performed ECT-treatments. Furthermore, about 50% of the responding Ger and Sui forensic psychiatric hospitals reported a high need for ECT by rating the question with at least 8 out of 10 points on the numeric scale (see Fig. 1). In our previous study, only 37% of the responding Ger forensic hospitals reported a high need for ECT ≥ 8, using the same scale [16].

The promising effectiveness of ECT in the reported forensic patients, even in the context of the limited total number of treated patients, can be considered remarkable. With response rates of 55.2% (Ger) and 50.0% (Sui) these are comparable to those in patients with schizophrenia treated with ECT in general psychiatric hospitals [12, 19–21]. At this point it must be emphasized, that nearly all reported patients (Ger = 86.2%; Sui = 100%) suffered from treatment resistant schizophrenia, thus largely lacking other treatment options beside of ECT.

Despite of the doubled indication rate, higher estimated need for ECT in forensic psychiatry and the promising effectiveness, the number of patients treated with ECT in forensic psychiatric hospitals in Ger (36 patients) and Sui (2 patients) is still very low. Even after the updated S3-guideline for schizophrenia from 2019 and the included new recommendation for the use of ECT in patients with antipsychotic treatment-resistance [17], we could only identify four more ECT-treatments in forensic hospitals in Ger compared to our previous study of 2018 [16]. Less than half of the forensic hospitals (Ger: 44.8%) with the possibility to offer their patients ECT reported a treatment in the assessed period. However, due to the slightly lower reply rate to our extended questionnaire (Ger: 2018 = 65.8%; 2024 = 52.6%), the actual increase in treatments may be slightly larger.

Like in 2018 [16], lacking infrastructure (*n* = 9 out of 12; 75%) still is– by far– the most important reason why ECT may not be performed in a Ger forensic hospital. Ethical concerns, on the other hand, were only mentioned in one case here (2018: 22.7%) [16]. This might be a result of the growing literature regarding the use and effectivity of ECT in the most severely affected patient groups, including those lacking decision-making capacity [22]. Moreover, several publications from an ethical perspective stated that any restrictions of patients’ access to ECT is ethically problematic [23, 24].

In the light of several supreme court decisions concerning the use of ECT in incapable patients who refused ECT– in Ger as well as in Sui– it seems surprising that only two responding hospitals from Ger and none from Sui chose legal obstacles as hindering reason for not performing ECT [25–27]. Yet, the different and complex court decisions might explain, why only half of the responding hospitals from Ger (51.7%) and Sui (50.0%) would consider ECT against a patient’s will, although studies demonstrated that ECT can be highly effective in incapable patients [16, 22].

In contrast to the forensic hospitals in Ger, all four responding clinics from Sui declared to have the possibility to perform ECT. Nevertheless, only 25.0% of the forensic hospitals in Sui reported a treatment with ECT in the respective period. This restraint counteracts the general development in Sui general psychiatric hospitals: the availability of ECT has clearly increased over the past decade [14].

From a medical ethics perspective, the promising effectiveness of ECT in forensic patients raises the question, why those severely ill patients seem not to have equal access to ECT, compared with patients in general psychiatry. While a huge proportion of forensic patients suffer from treatment resistant schizophrenia, ECT might be their only chance for a relevant reduction of symptoms, restored decision-making capacity and, as a result, progress in the easing of restraints and detention. Conversely, a lack of treatment with ECT could force the respective patient to be detained in a forensic psychiatric hospital for a long and potentially unnecessary period. In view of this, the lack of equal access to ECT for forensic psychiatric patients cannot be ethically justified.

## Limitations

One main limitation derives from the retrospective nature of our study: post hoc assessments are generally more prone to confounding factors (memory bias) than prospective designs [28]. Furthermore, we cannot rule out that hospitals performing ECT or those with an overall positive attitude towards ECT may have been overrepresented in our study in the form of a selection bias. Despite the high response rate of 52.5% (Ger) and 66.7% (Sui), our findings may thus not be generalizable on the entirety of forensic psychiatric hospitals in both countries: Forensic hospitals without ECT might have been less interested to answer a questionnaire investigating this topic. However, we also point out that the extent of our questionnaire might have discouraged hospitals with a large number of ECT treatments, as the required time to complete the questionnaire increased with the number of patients– this would in turn counteract a selection bias. In future studies on this topic, the involvement of regulating authorities might be helpful to further reduce the reporting bias by motivating more forensic hospitals to participate in the survey.

## Conclusion

There seems to be a better awareness of the (potential) effectiveness of ECT in patients with schizophrenia and related disorders in forensic settings, represented by the doubled indication rate and higher estimated need for ECT in forensic psychiatry found here. Our retrospective study shows the potential benefits for these patients in terms of both symptom decrease and reduction of necessary restraints, even in the context of the limited data available. Nevertheless, there still seem to be barriers for the wider implementation of indicated ECT treatments in forensic psychiatry, mostly lacking infrastructure. From a medical ethics perspective, further measures should be taken to facilitate forensic patients’ access to ECT (structural requirements, where necessary via cooperation with other clinics, possible legal restrictions in patients lacking decision making capacity). Furthermore, it would be of great importance to confirm the limited evidence for the effectiveness of ECT in forensic settings, mostly derived from case reports, case series and retrospective studies [15], by prospective controlled observational studies.

## Data Availability

The data that support the findings of this study are available from the corresponding author upon reasonable request.
